# Association of Vitamin D Receptor Polymorphism with Susceptibility to Symptomatic Pertussis

**DOI:** 10.1371/journal.pone.0149576

**Published:** 2016-02-19

**Authors:** Wanda G. H. Han, Hennie M. Hodemaekers, Bhawani Nagarajah, Martien M. C. Poelen, Kina Helm, Riny Janssen, Cécile A. C. M. van Els

**Affiliations:** 1 Centre for Infectious Disease Control, National Institute for Public Health and the Environment, Bilthoven, the Netherlands; 2 Centre for Health Protection, National Institute for Public Health and the Environment, Bilthoven, the Netherlands; University of Birmingham, UNITED KINGDOM

## Abstract

Pertussis, caused by infection with the gram negative *B*. *pertussis* bacterium, is a serious respiratory illness that can last for months. While *B*. *pertussis* infection rates are estimated between 1–10% in the general population, notifications of symptomatic pertussis only comprise 0.01–0.1% indicating that most individuals clear *B*. *pertussis* infections without developing (severe) clinical symptoms. In this study we investigated whether genetic risk factors are involved in the development of symptomatic pertussis upon *B*. *pertussis* infection. Single-nucleotide polymorphisms (SNPs) in candidate genes, *MBL2*, *IL17A*, *TNFα*, *VDR*, and *IL10* were genotyped in a unique Dutch cohort of symptomatic clinically confirmed (ex-)pertussis patients and in a Dutch population cohort. Of the seven investigated SNPs in five genes, a polymorphism in the Vitamin D receptor (VDR) gene (rs10735810) was associated with pertussis. The *VDR* major allele and its homozygous genotype were more present in the symptomatic pertussis patient cohort compared to the control population cohort. Interestingly, the *VDR* major allele correlated also with the duration of reported pertussis symptoms. Vitamin D_3_ (VD_3_) and VDR are important regulators of immune activation. Altogether, these findings suggest that polymorphisms in the *VDR* gene may affect immune activation and the clinical outcome of *B*. *pertussis* infection.

## Introduction

*Bordetella (B*.*) pertussis* is the causative agent of pertussis (whooping cough), an acute infection of the upper respiratory tract. The clinical manifestations of pertussis are characterized by three phases: the catarrhal phase, paroxysmal phase and convalescent phase [1, 2]. In the catarrhal phase, the symptoms are usually mild, similar to those of the common cold. After 1–2 weeks, classic symptoms of pertussis, such as paroxysmal cough, inspiratory whoop and vomiting, develop. Recurrence of paroxysmal coughing fits can persist for weeks to months after the infection has been cleared. In the convalescent phase, symptoms decline gradually over another month. In infants, *B*. *pertussis* infection can result in pneumonia and in some cases respiratory failure and death. Introduction of pertussis vaccination, since the 1950’s in many western countries, has reduced the incidence of pertussis and the number of deaths caused by infection. However, despite high vaccination coverage, pertussis is still observed not only in non- or partially vaccinated infants but also adolescent, adult and elderly vaccinees, and the incidence is increasing in the last decades [3]. Thus, although vaccination strategies have reduced pertussis morbidity in childhood, pertussis remains a serious respiratory illness that can last for months.

The incidence of pertussis in the vaccinated population based on clinical notifications is estimated between 0.01–0.1% in USA [4], also applying for the Netherlands in the period 1996–2012 [5]. However based on a large Dutch serosurveillance study in 2006–07, 9.3% of the population older than 9 years had elevated *B*. *pertussis*-specific antibody levels, indicative for *B*. *pertussis* infection in the past year. Thus *B*. *pertussis* infection seems quite common in the general population yet only a minority of infected individuals develops symptomatic pertussis. What determines whether *B*. *pertussis* infected individuals control the infection with or without displaying (severe) clinical symptoms? Besides environmental factors (vaccination history and pathogen exposure), host genetic polymorphisms may result in different innate and adaptive immune responses to a pathogen, and subsequent differential susceptibility to disease [6]. To identify possible genetic risk factors for developing symptomatic pertussis we benefitted from a unique cohort of symptomatic clinically confirmed (ex-)pertussis patients with known reported symptoms to perform a case-population cohort genetic association study.

We included for this study 195 Dutch clinically confirmed (ex-)pertussis cases in the pertussis patient cohort and 462 controls, representative for the general Dutch population, in the control population cohort. Based on literature, candidate SNPs in the *MBL2*, *IL17A*, *TNFα*, *VDR* and *IL10* genes were selected for investigation. Mannan-binding lectin (MBL) is critical for complement activation via the lectin pathway, thus having an important anti-microbial function. Three SNPs in the *MBL2* gene, (allele D; rs5030737, allele B; rs1800450, and allele C; rs1800451) cause reduced MBL serum levels [7, 8]. MBL deficiency measured in serum has been associated with susceptibility to certain infectious diseases including pertussis [9, 10]. A recent study did not find an association between MBL polymorphisms and antibody responses after acellular pertussis vaccinations [8]. IL-17A is a characteristic cytokine for Th17 immunity and Th17 type CD4^+^ T cells play an important role in the protective immune response against *B*. *pertussis* [11]. The rs2275913 SNP in the *IL17A* gene affects IL-17A production in PBMC [12]. Furthermore, an association of the SNP with respiratory diseases, such as asthma and bronchiolitis, has been shown [12]. TNFα is an important innate and adaptive pro-inflammatory cytokine. The rs1800629 SNP in the *TNFα* gene affects TNFα production in PBMC [13]. Also an association between the SNP and RSV bronchiolitis and asthma [14] and vaccine-specific immune response [15] was found. Vitamin D receptor (VDR) mediates biological action of Vitamin D_3_ (VD_3_) and VD_3_ has been shown to have immunomodulatory properties [16]. The rs10735810 SNP in the *VDR* gene affects VDR signaling in immune cells [17]. Moreover, the SNP has been associated with severe RSV bronchiolitis and tuberculosis [18, 19]. IL-10 is an important anti-inflammatory cytokine. The rs1800872 SNP affects IL-10 production in stimulated PBMC [20]. Furthermore, severe RSV bronchiolitis was associated with this SNP [14, 18]. The pertussis patient cohort provided unique material to investigate whether polymorphisms in these candidate genes are involved in the development of symptomatic pertussis upon *B*. *pertussis* infection.

## Materials and Methods

### Study populations

Volunteers were recruited in accordance with principles expressed in the Declaration of Helsinki and with Dutch regulations in two clinical studies. A pertussis patient cohort of 195 persons (86 males/109 females) consisted of symptomatic pertussis (ex-)cases with laboratory (i.e. PCR, culture or serodiagnostics) confirmed *B*. *pertussis* infection, who were included at varying time intervals after their date of diagnosis (average 36.5 months, range 0.3–450.9 months) in the SKI study (CCMO nr: NL16334.040.07). This SKI study was performed in the Netherlands between 2008 and 2012 after approval by the accredited Review Board STEG (Stichting Therapeutisch Evaluatie Geneesmiddelen) and METC UMC Utrecht (Medisch Ethische Toetsingscommissie Universitair Medisch Centrum Utrecht), and after approval of practicability by the Review Boards of collaborating hospitals. A control population cohort of 462 persons (208 males/254 females) was randomly taken from the Regenboog study [21], a large Dutch population health examination survey and approved by the local review board of the RIVM in 1998. All participants, or both parents in case of minor participants, included in both studies provided written informed consent for the collection and the described use of samples and the usage of a completed questionnaire. For participants in the pertussis patient cohort this questionnaire included questions regarding clinical parameters such as type and duration of pertussis symptoms, medication and vaccination history.

### Clinical samples

For the control population cohort, venous blood samples were collected in citrated tubes and buffy coat cells were isolated using standard procedures and stored at -20°C. For the pertussis patient cohort, venous blood samples were collected in vacutainer cell preparation (CPT) tubes (BD), peripheral blood mononuclear cells (PBMC) were isolated using standard procedures and B lymphoblastoid cell lines (B-LCL) were generated from a small aliquot of PBMC after transformation with B95-8 Epstein Barr Virus (EBV) supernatant.

### Selection of genes and SNPs

Literature search for candidate genes and SNPs involved in symptomatic pertussis was performed by using HuGE Navigator (version 2.0), an integrated, searchable knowledge base of genetic associations and human genome epidemiology [22]. The selection of five candidate genes and seven SNPs was based on relevance for pertussis and associated terms such as anti-microbial, respiratory disease and vaccination response. SNPs with minor allele frequency of <5% were excluded, considering the sample size. For MBL, genotypes were categorized in A/A as the wildtype, A/O as the heterozygotes variants and O/O as the homozygotes variants in which O stands for any variant alleles B (rs1800450), C (rs1800451) or D (rs5030737) [8]. All selected SNPs in *MBL2*, *IL17A*, *TNFα*, *VDR* and *IL10*, influence the function of the respective genes.

### DNA isolation and genotyping

For the pertussis patient cohort, DNA was isolated from cryopreserved B-LCL using the QIAamp DNA Blood Mini Kit (Qiagen). For the control population cohort, DNA was isolated as described elsewhere [23]. The DNA concentration was determined using a NanoDrop spectrophotometer (Isogen Life Science, De Meern, the Netherlands). SNPs were genotyped using KASP genotyping technology by LGC Genomics (Hoddesdon, UK). Genotyping was visualized in SNPviewer version 1.98 (LGC Genomics, Hoddesdon, UK) and results for 46 genotyping datapoints were excluded because of a low signal, overlapping or multiple clusters, or scattering of the clusters.

### Statistical analysis

Data analyses were performed using SPSS Statistics version 22 (IBM). Genotype frequencies for pertussis patient and control population cohorts were in Hardy-Weinberg equilibrium, except for *MBL2* rs5030737 (P<0.001). Disease association with each polymorphism was analysed by 2 x 2 (allele distribution) or 2 x 3 (genotype distribution) Pearson Chi-Square tests.

## Results

### Characteristics of symptomatic pertussis patients

In total, 195 Dutch pertussis patients were involved in the study (Table 1). The majority of the pertussis patient cohort experienced symptomatic pertussis during childhood (17.3%, 12.8% and 34.7% as 0, 1–4, 5–14 year olds, respectively), in line with Dutch epidemiological data [5]. Most (84.2%) patients had received pertussis vaccination (whole cell pertussis vaccine (wP) (75.4%), acellular pertussis vaccine (aP) (7.7%) or both wP and aP (1.0%)). 31 patients (15.9%) had no history of pertussis vaccination since they were born before the introduction of the pertussis vaccine in the Dutch National Immunization Program (n = 23) or because they were voluntary non-vaccinated (n = 8). Thus, 95.3% of the patient cohort eligible for the Dutch National Immunization Program did participate which is in line with the vaccination coverage in the Dutch population (92–99%) [24]. In all the pertussis patients, of which information on the type of symptoms was available, at least one of the characteristic symptoms such as coughing spells with wheezing, thick mucus, vomiting or chronic coughing was noted. In half of the patients (53.6%), symptoms persisted longer than 8 weeks.

**Table 1:** Characteristics of symptomatic pertussis patients.

	N	(%)
**Cases**	195	
**Male/female**	87/109	(44%/56%)
**Age at symptomatic pertussis infection**		
0 years old	34	(17.3%)
1–4 years old	25	(12.8%)
5–14 years old	68	(34.7%)
15–24 years old	10	(5.1%)
25–44 years old	17	(8.7%)
45–64 years old	30	(15.3%)
65–74 years old	10	(5.1%)
75+	1	(0.5%)
**History of pertussis vaccination**		
Whole cell pertussis vaccine (wP)	147	(75.4%)
Acellular pertussis vaccine (aP)	15	(7.7%)
Both wP and aP	2	(1.0%)
No vaccination^a^	31	(15.9%)
**Clinical symptoms**^b^		
Coughing spells with wheezing	151	(79.1%)
Thick mucus	137	(71.7%)
Vomiting	109	(57.1%)
Chronic coughing	154	(80.6%)
**Duration of symptoms**^c^		
< 4 weeks	15	(7.7%)
4–8 weeks	74	(37.8%)
> 8 weeks	105	(53.6%)

^a^ 23x born before introduction of pertussis vaccine in Dutch National Immunization Program, 8x voluntary non-vaccinated

^b^ 4x missing data

^c^ 1x missing data

### Vitamin D receptor polymorphism association with symptomatic pertussis

The selected candidate SNPs, were genotyped for the symptomatic pertussis patient cohort (n = 195) and the control population cohort (n = 462) (Table 2). Of the seven investigated SNPs, the genotype distribution was significantly different for the *VDR* SNP (p.Thr1Met; rs10735810) between patient and control cohort (p = 0.039) (Table 2). A higher percentage of symptomatic pertussis patients (50.0%) possessed the homozygous G:G genotype compared to the control group (39.2%), OR 1.55 (Table 2). A lower percentage of patients (39.6%) was found to present the heterozygous (G:A) genotype compared to the control group (47.7%), OR 0.72 (Table 2). In accordance with the *VDR* genotype association, the allele frequency of the *VDR* major allele occurred at a higher frequency in the pertussis patient group (69.8%) compared to the control population group (63.1%), p = 0.020 and OR 1.35 (Table 3). The minor allele of the *TNFα* SNP (c.308G>A; rs1800629) was present at a higher frequency in the pertussis patient group (19.8%) compared to the control population group (16.7%) (Table 3), however the difference was not significant. The frequencies of the other investigated SNPs, *IL17A*, *MBL2* and *IL10*, were similar in patient and control group (Tables 2 and 3). When correcting for multiple testing of five investigated genes, the *VDR* SNP genotype and allele distribution p-values were not significant (p = 0.20 and p = 0.10, respectively). Altogether, these data indicate that the *VDR* major allele is weakly associated with symptomatic pertussis.

**Table 2:** Genotype frequencies and OR for the *VDR*, *TNFα*, *IL17*, *MBL2* and *IL10* SNPs in the pertussis patient group and control group.

Gene	rs number	SNP	Genotype	Patient group n = 195^a^	Control group n = 462^b^	P^c^	OR (95% CI)
*VDR*	rs10735810	p.Thr1Met	G:G	96 (50.0%)	180 (39.2%)	**.039** (.20^d^)	1.55 (1.10–2.18)
			G:A	76 (39.6%)	219 (47.7%)		0.72 (0.51–1.01)
			A:A	20 (10.4%)	60 (13.1%)		0.77 (0.45–1.32)
*TNFα*	rs1800629	c.308G>A	G:G	122 (63.2%)	319 (70.1%)	.170	
			G:A	65 (33.7%)	120 (26.4%)		
			A:A	6 (3.1%)	16 (3.5%)		
*IL17A*	rs2275913	c.152G>A	G:G	87 (45.3%)	211 (46.4%)	.578	
			G:A	86 (44.8%)	188 (41.3%)		
			A:A	19 (9.9%)	56 (12.3%)		
*MBL2*	rs1800450/ rs1800451/ rs5030737	p.Gly54Asp/ p.Gly57Glu/ p.Arg52Cys	A/A	116 (60.1%)	269 (59.4%)	.887	
			A/O	68 (35.2%)	166 (36.6%)		
			O/O	9 (4.7%)	18 (4.0%)		
*IL10*	rs1800872	c.592C>A	C:C	122 (63.2%)	290 (63.3%)	.999	
			A:C	62 (32.1%)	147 (32.1%)		
			A:A	9 (4.7)	21 (4.6%)		

**Note.** OR, odds ratio; CI, confidence interval

^a^ SNPs undetected in 3 individuals for *VDR*, 2 for *TNFα*, 3 for *IL17A*, 1 for *MBL2* (rs1800450), 1 for *MBL2* (rs1800451), 2 for *MBL2* (rs5030737), and 2 for *IL10* in patient group.

^b^ SNPs undetected in 3 individuals for *VDR*, 7 for *TNFα*, 7 for *IL17A*, 1 for *MBL2* (rs1800450), 1 for *MBL2* (rs1800451), 9 for *MBL2* (rs5030737), and 4 for *IL10* in control group.

^c^ According to Pearson Chi-Square distribution of a 2 x 3 table on genotype frequencies.

^d^ Bonferroni correction-adjusted P-value.

**Table 3:** Allele frequencies and OR for the *VDR*, *TNFα*, *IL17*, *MBL2* and *IL10* SNPs in the pertussis patient group and control group.

Gene	rs number	SNP	Allele	Patient group n = 195^a^	Control group n = 462^b^	P^c^	OR (95% CI)
*VDR*	rs10735810	p.Thr1Met	G	268 (69.8%)	579 (63.1%)	**.020** (.10^d^)	1.35 (1.05–1.75)
			A	116 (30.2%)	339 (36.9%)		0.74 (0.57–0.95)
*TNFα*	rs1800629	c.308G>A	G	309 (80.1%)	758 (83.3%)	.161	
			A	77 (19.9%)	152 (16.7%)		
*IL17A*	rs2275913	c.152G>A	G	260 (67.7%)	610 (67.0%)	.813	
			A	124 (32.3%)	300 (33.0%)		
*MBL2*	rs1800450	p.Gly54Asp	G	336 (86.6%)	807 (87.5%)	.645	
			A	52 (13.4%)	115 (12.5%)		
	rs1800451	p.Gly57Glu	G	379 (97.7%)	903 (97.8%)	.767	
			A	9 (2.3%)	19 (2.1%)		
	rs5030737	p.Arg52Cys	C	355 (92.0%)	824 (90.9%)	.553	
			T	31 (8.0%)	82 (9.1%)		
*IL10*	rs1800872	c.592C>A	C	306 (79.3%)	727 (79.4%)	.970	
			A	80 (20.7%)	189 (20.6%)		

**Note.** OR, odds ratio; CI, confidence interval

^a^ SNPs undetected in 3 individuals for *VDR*, 2 for *TNFα*, 3 for *IL17A*, 1 for *MBL2* (rs1800450), 1 for *MBL2* (rs1800451), 2 for *MBL2* (rs5030737), and 2 for *IL10* in patient group.

^b^ SNPs undetected in 3 individuals for *VDR*, 7 for *TNFα*, 7 for *IL17A*, 1 for *MBL2* (rs1800450), 1 for *MBL2* (rs1800451), 9 for *MBL2* (rs5030737), and 4 for *IL10* in control group.

^c^ According to Pearson Chi-Square distribution of a 2 x 2 table on allele frequencies.

^d^ Bonferroni correction-adjusted P-value.

### Correlation duration of symptoms and VDR polymorphism

The pertussis patient group included individuals with symptomatic pertussis, however within this cohort subgroups can be identified based on clinical parameters (Table 1). We found no significant differences in VDR allele and genotype distribution when the patient cohort was subgrouped on sex, age at symptomatic pertussis, history of pertussis vaccination, clinical symptoms (data not shown). When pertussis patients were subgrouped for the reported duration of symptoms (< 4 weeks, 4–8 weeks and > 8 weeks), *VDR* SNP genotype and allele distribution was significantly different (p = 0.002 and p = 0.033, respectively) (Table 4). A higher percentage of symptomatic pertussis patients with a duration of symptoms > 8 weeks (53.4%) possessed the homozygous G:G genotype compared to the patient subgroup with a duration of symptoms of 4–8 weeks (47.9%) and < 4 weeks (40.0%). In line with this observation, the *VDR* major allele occurred at a higher frequency in the patient subgroup with a duration of symptoms of > 8 weeks (73.3%) compared to the patient subgroup with a duration of symptoms of 4–8 weeks (69.9%) and < 4 weeks (63.1%). When the pertussis patients with a duration of symptoms > 4 weeks were selected for *VDR* polymorphism distribution analysis (n = 176) compared with control group (n = 462), the association of the G:G genotype with symptomatic pertussis was increased (p = 0.012) (Table 5) compared to the total pertussis patient cohort (p = 0.046) (Table 2). Moreover, a strong association of the VDR major allele (G) was found in symptomatic pertussis patients with a duration of symptoms > 4 weeks (p = 0.003) (Table 5) and this association was significant after correcting for multiple testing (p = 0.015). Thus, the *VDR* major allele and its homozygous genotype are associated with symptomatic pertussis and in addition correlate with the duration of symptoms.

**Table 4:** Distribution of *VDR* genotypes and alleles in the pertussis patient group according to the reported duration of symptoms.

Duration of symptoms Total n = 191^c^	*VDR* genotype^a^			*VDR* allele^b^	
	G:G	G:A	A:A	G	A
< 4 weeks (n = 15)	6 (40.0%)	3 (20.0%)	6 (40.0%)	15 (50.0%)	15 (50.0%)
4–8 weeks (n = 73)	35 (47.9%)	32 (43.8%)	6 (8.2%)	102 (69.9%)	44 (30.1%)
> 8 weeks (n = 103)	55 (53.4%)	41 (39.8%)	7 (6.8%)	151 (73.3%)	55 (26.7%)

^a^ P = 0.002 according to Pearson Chi-Square distribution of a 3 x 3 table on *VDR* SNP genotype frequencies.

^b^ P = 0.033 according to Pearson Chi-Square distribution of a 2 x 3 table on *VDR* SNP allele frequencies.

^c^ *VDR* SNPs undetected in 3 patients and for 1 patient ‘duration of symptoms’ data was missing.

**Table 5:** Association *VDR* SNP with pertussis in patients with symptoms that persisted for longer than 4 weeks.

*VDR*	SNP typing	Patient group n = 176^a^	Control group n = 459^b^	P^c^	OR (95% CI)
Genotype level	G:G	90 (51.1%)	180 (39.2%)	**.012** (.06^d^)	1.62 (1.14–2.30)
	G:A	73 (41.5%)	219 (47.7%)		0.78 (0.55–1.10)
	A:A	13 (7.4%)	60 (13.1%)		0.53 (0.28–0.99)
Allele level	G	253 (71.9%)	579 (63.1%)	**.003** (**.015**^d^)	1.50 (1.14–1.96)
	A	99 (28.1%)	339 (36.9%)		0.67 (0.51–0.87)

**Note.** OR, odds ratio; CI, confidence interval

^a^ SNPs undetected in 3 individuals for *VDR* in patient group. Missing duration of symptoms data for 1 individual. 15 pertussis patients reported a duration of symptoms < 4 weeks.

^b^ SNPs undetected in 3 individuals for *VDR* in control group.

^c^ According to Pearson Chi-Square distribution of 2 x 3 or 2 x 2 table on genotype or allele frequencies, respectively.

^d^ Bonferroni correction-adjusted P-value.

## Discussion

To identify genes important for susceptibility to symptomatic pertussis, we performed a case-population cohort genetic association study comparing the distribution of candidate SNPs in a symptomatic pertussis patient cohort and a control population cohort. In this study, we found an association of the *VDR* major allele (rs10735810) and its homozygous genotype with symptomatic pertussis (Tables 2 and 3). Interestingly, the *VDR* major allele (G) correlated also with the duration of pertussis symptoms (Table 4). When the pertussis patients with a duration of symptoms < 4 weeks were excluded, the association of the G:G genotype and G allele with symptomatic pertussis increased (Table 5). The number of symptomatic pertussis patients with a duration of symptoms < 4 weeks was small in our patient group (n = 15) and seemed to contain an overrepresentation of 0 and 1–4 year olds (40.0% and 26.7%, respectively) (data not shown) compared to the total pertussis patient group (17.4% and 12.8%, respectively) (Table 1). It is likely that infants and young children with pertussis symptoms will visit their doctor at earlier notice than older children and adults, resulting in this bias. Also 6 out of 15 patients (40%) with symptoms < 4 weeks had received acellular pertussis vaccination compared to 7.7% in the total patient group (Table 1). Moreover, a lower percentage of symptomatic pertussis patients with a duration of symptoms < 4 weeks experienced vomiting (26.7%) (data not shown) compared to the total patient group (55.6%) (Table 1). Furthermore, no other clinical parameters such as sex, history of pertussis vaccination, underlying hypersensitivities (asthma, allergies, etc), were significantly different between the patient groups with a duration of symptoms of < 4 weeks, 4–8 weeks and >8 weeks (data not shown). However, these findings indicate that the pertussis patient cohort in our study is not a homogenous population, and focusing on the more severe patients with persisting symptoms may enhance the observed effect. Altogether, these findings suggest that the *VDR* SNP influences the clinical outcome of *B*. *pertussis* infection.

The vitamin D receptor (VDR) mediates largely the biological actions of VD_3_. The level of available VD_3_ is dependent on dietary uptake, synthesis in the skin upon UV-B exposure and vitamin D-binding protein (VDBP) concentration and binding affinity [25, 26]. VD_3_ is hydroxylated in the liver to 25(OH)VD_3_, the main circulating form, and mostly bound to VDBP. Subsequently, 25(OH)VD_3_ is metabolized in the kidneys or locally in tissues by immune cells to the most physiologically active VD_3_ metabolite, 1,25(OH)_2_VD_3_. 1,25(OH)_2_VD_3_-liganded VDR can directly induce gene transcription by interacting with vitamin D-responsive elements (VDRE) in the promoter region of target genes involved in processes such as calcium absorption and bone homeostasis, immune, central nervous and endocrine systems and epithelial cell differentiation [27]. For example 1,25(OH)_2_VD_3_ induces antimicrobial peptide gene expression in neutrophils, monocytes and epithelial cells, contributing to immediate innate microbial clearance mechanisms [26, 28]. At the same time, 1,25(OH)_2_VD_3_-liganded but also unliganded VDR can interfere with the instruction of an adaptive immune response, by down-regulating the signaling of transcription factors such as NF-κB and exerting anti-inflammatory effects [26, 29]. 1,25(OH)_2_VD_3_ can suppress maturation and differentiation of antigen-presenting cells (APC), due to downregulation of pattern recognition receptors (PRR) such as TLR2, TLR4 and TLR9 [30, 31]. Moreover, 1,25(OH)_2_VD_3_ can through modulation of the APC, but also directly, skew T cell differentiation toward Th2 and Treg at the expense of pro-inflammatory Th1 and Th17 [32–34]. Hence, VD_3_ has both anti-microbial and anti-inflammatory effects in infectious diseases [26]. Various functional SNPs in *VDR* have been described and are usually referred to by the names of digestion enzymes used for genotyping (*FokI*, *BsmI*, *ApaI*, *and TaqI*) [35]. The polymorphisms *BsmI*, *ApaI* and *TaqI* are located in the 3’ untranslated region, and the *FokI* polymorphism in the coding region. The *VDR* rs10735810 *FokI* SNP investigated in this study has structural implications, resulting in either a long VDR variant or a three amino acids shorter VDR version (minor and major allele, respectively). Several studies show functional consequences of this SNP for VDR activity [17, 27]. The differential outcome of the VDR polymorphism on the function of the VDR receptor to dampen the pro-inflammatory cytokine response can be measured by incubating primary cells from VDR typed individuals with pro-inflammatory stimuli in the presence or absence of 1,25(OH)_2_VD_3_. Van Etten *et al*. showed that while 1,25(OH)_2_VD_3_ mediated down-regulation of IL-12 expression was in place in both individuals homozygous for the major or minor VDR allele, the net expression of IL-12 by activated human monocytes and dendritic cells from VDR major allele individuals was higher compared to VDR minor allele individuals [17]. Hence, VD_3_ can exert its anti-inflammatory effects through both *FokI* major and minor VDR, yet the major VDR variant is associated with a higher overall pro-inflammatory immune status. The biological consequences of the other VDR polymorphisms are less clear.

The pathogenesis of pertussis remains unclear due to the lack of animal models that resemble human pathology after infection with *B*. *pertussis* [2, 36]. *B*. *pertussis* virulence factors, such as pertussis toxin and lipo-oligosaccharide (LOS), are thought to attribute to the disease [2, 37]. Via engaging the innate TLR4 pathway of the host, LOS is an important driver of the pro-inflammatory and subsequent Th1/Th17 response, effective against *B*. *pertussis* [38]. However, LOS can also induce excessive immunological responses, illustrated by the reactogenicity of first generation whole cell pertussis vaccines containing LOS [39]. Thus although a pro-inflammatory response is essential for the clearance of *B*. *pertussis* [11, 40], inflammatory lung pathology should be prevented [41]. In our symptomatic pertussis patients, an increase in individuals possessing the more pro-inflammatory major VDR (G:G) genotype was found compared to the control population. A higher overall pro-inflammatory immune status in these patients might have contributed to inflammatory lung pathology and persistent clinical symptoms. Interestingly, the *VDR* SNP rs10735810 has also been shown to be associated with susceptibility to tuberculosis [19] and RSV bronchiolitis in children [18, 42, 43]. In tuberculosis (TB), associations with both the minor and major allele of the VDR SNP were found in different studies [19]. Moreover, the combination of VDR genotype and 25(OH)VD_3_ serum level seemed to affect clinical outcome in tuberculosis [44]. In the RSV bronchiolitis study and contrasting to our study, however, the less pro-inflammatory minor allele of the *VDR* SNP occurred at a higher frequency in the RSV patients’ cohort compared to the control population group. Although RSV bronchiolitis and pertussis are both respiratory infectious diseases, the pathogenic mechanisms may be very different. Unlike pertussis pathology, clinical manifestations of RSV bronchiolitis are mainly due to Th2-mediated immune pathology, thus a less pro-inflammatory hence more Th2-prone immune system would be detrimental in RSV bronchiolitis [45]. The different pathogenic mechanisms in pertussis, TB and RSV bronchiolitis may explain in part the opposing VDR SNP associations found with host susceptibility to respiratory disease.

Besides *FokI*, also *BsmI* polymorphism in VDR has been associated with host susceptibility to TB [19]. Thus, the functionality of the VDR may not be fully appreciated by genotyping the *FokI* polymorphism alone. It has been hypothesized that one or more unknown additional SNPs in *VDR* beyond *FokI*, *BsmI*, *ApaI* and *TaqI*, affect VDR activity and expression [35]. In addition, the TB related VDR SNPs were found in an Asian population study, yet not in Africans or South Americans [19]. Ethnic-specific genetic or cultural factors may enhance or abolish the effects of VDR polymorphisms on host susceptibility to respiratory diseases such as TB. Also, it would be interesting to combine the results of VDR genotyping with measurements of 25(OH)VD_3_ levels in patients’ sera, perhaps enforcing the observed VDR SNP association. Unfortunately, blood samples of the symptomatic pertussis patient cohort were collected at widely varying time points after diagnosis and during different seasons of the year. Since the VD_3_ serum levels are dependent on environmental factors such as diet and exposure to (seasonal) sunlight [46], we cannot investigate whether the 25(OH)VD_3_ serum levels in pertussis patients at the time of disease would have influenced the VDR SNP association with symptomatic pertussis. Moreover, genetic polymorphisms in other mediators in the VD_3_ pathway, such as VDBP and hydroxylases [47], also impact bioavailable 25(OH)VD_3_ levels and VDBP haplotypes were associated with host susceptibility to RSV bronchiolitis [48]. Thus, the interplay of VD_3_ serum levels, genetic polymorphisms in VDR and other mediators in the VD_3_ pathway would together determine the VD_3_ status of the individual and subsequently the impact on the immune response upon infection with a pathogen, rather than the investigated VDR *FokI* polymorphism alone. Due to the complexity of the immune system, multiple redundancies, interfering signaling pathways, environmental factors and distinct pathogenic mechanisms for different pathogens, the clinical impact of the *VDR* SNP may have differential and even opposite effects which remain to be further elucidated.

In summary, our results show that the *VDR* gene *Fok*I polymorphism, associated with a higher pro-inflammatory immune status, is associated with symptomatic pertussis. Moreover, a correlation of the *VDR* major allele with the duration of clinical symptoms was found. The impact of *FokI* and other VDR polymorphisms on the pathogenesis of symptomatic pertussis needs further investigation. Nevertheless, these findings highlight the importance of VD_3_ effects on the immune system and that the delicate balance of pro- and anti-inflammatory immune mechanisms may determine the clinical outcome of *B*. *pertussis* infection.
